# TMT-based quantitative proteome data of MSP1 overexpressed rice

**DOI:** 10.1016/j.dib.2022.108791

**Published:** 2022-11-29

**Authors:** Cheol Woo Min, Jeong Woo Jang, Gi Hyun Lee, Ravi Gupta, Sun Tae Kim

**Affiliations:** a Department of Plant Bioscience, Life and Industry Convergence Research Institute, Pusan National University, Miryang 50463, South Korea; b College of General Education, Kookmin University, Seoul 02707, South Korea

**Keywords:** Rice blast disease, Plant-pathogen interaction, PRRs, Proteomics

## Abstract

Data reported here is associated with the article entitled “TMT-based quantitative membrane proteomics identified pattern recognition receptors (PRRs) potentially involved in the perception of MSP1 in rice leaves” [1]. PAMP-triggered immunity (PTI) constitutes the first layer of plant innate immunity against pathogen infection. *M. oryzae* secreted protein MSP1 has been identified as a PAMP which induces PTI responses in rice. However, identification of PRRs involved in the recognition of MSP1 has not been achieved so far. In this manuscript, we carried out comprehensive proteomic profiling to investigate the potential PRRs and MSP1 induced signaling cascades using MSP1 overexpressed transgenic rice by TMT-labeling based quantitative analysis with QExactive^TM^ Orbitrap High-Resolution Mass Spectrometer [1].

**Specifications Table**

**Table:** 

Subject	Biology
Specific subject area	Plant science, Proteomics, Plant-pathogen interaction
Type of data	Table, Figure
How the data were acquired	QExactive™ Orbitrap High-Resolution Mass Spectrometer (Thermo Fisher Scientific, USA) coupled with UHPLC Dionex UltiMate^TM^ 3000 (Thermo Fisher Scientific, USA) systems
Data format	Raw, Analyzed
Description of data collection	Leaf soluble and microsomal membrane proteome of wild type (cv. Dongjin, DJ), extracellular MSP1 (eMSP1), and cytoplasmic MSP1 (cMSP1) overexpressed rice cultivars were analyzed with and without jasmonic acid (JA) treatment
Data source location	Plant Immunity Laboratory, Department of Plant Science, Pusan National University, Miryang, Republic of Korea (latitude 35 N)
Data accessibility	Repository name: ProteomeXchange Data identification number: PXD032689. Direct FTP link to data: http://proteomecentral.proteomexchange.org/cgi/GetDataset?ID=PXD032689 Repository name: Mendeley Data Data identification number: DOI:10.17632/ngh58j6c6w.1 Title: Supplementary files of ``TMT-based quantitative proteome data of MSP1 overexpressed rice'' Direct URL link to data: https://data.mendeley.com/datasets/ngh58j6c6w
Related research article	C.W. Min, J.W. Jang, G.H. Lee, R. Gupta, H.J. Park, H.S. Cho, S.R. Park, S. Kwon, L. Cho, K. Jung, Y. Kim, Y. Wang, S.T. Kim, TMT-based quantitative membrane proteomics identified PRRs potentially involved in the perception of MSP1 in rice leaves, J. Proteomics 267 (2022) 104687 [1].

## Value of the Data

• This data reports the potential pathogen associated molecular pattern (PAMP) recognition receptor (PRR) proteins from rice leaves involved in the identification of a *Magnaporthe oryzae* secreted MSP1 protein.
• Researcher's working on plant-pathogen interaction area can utilize abundance profile of 8,033 proteins to analyze the changes in abundance pattern of membrane-localized proteins in response to PAMP molecules.
• The list of plasma membrane localized-PRR(s) presented here can be use/re-used in the future studies to confirm their interaction with MSP1 in rice using biochemical and molecular biology approaches.

## 1 Objective

This work aimed to identify the potential PRRs involved in the recognition of a *M. oryzae* secreted PAMP, MSP1. In this manuscript, we report all the identified rice proteins of DJ, eMSP1, and cMSP1 rice plants identified using a TMT-based proteomics approach. While the original manuscript associated with this data report only 1826 significantly modulated proteins among DJ, eMSP1 and cMSP1 rice, here we report all the 8033 rice proteins including potential PRRs involved in MSP1 recognition.

## 2 Data Description

**Fig. 1:**
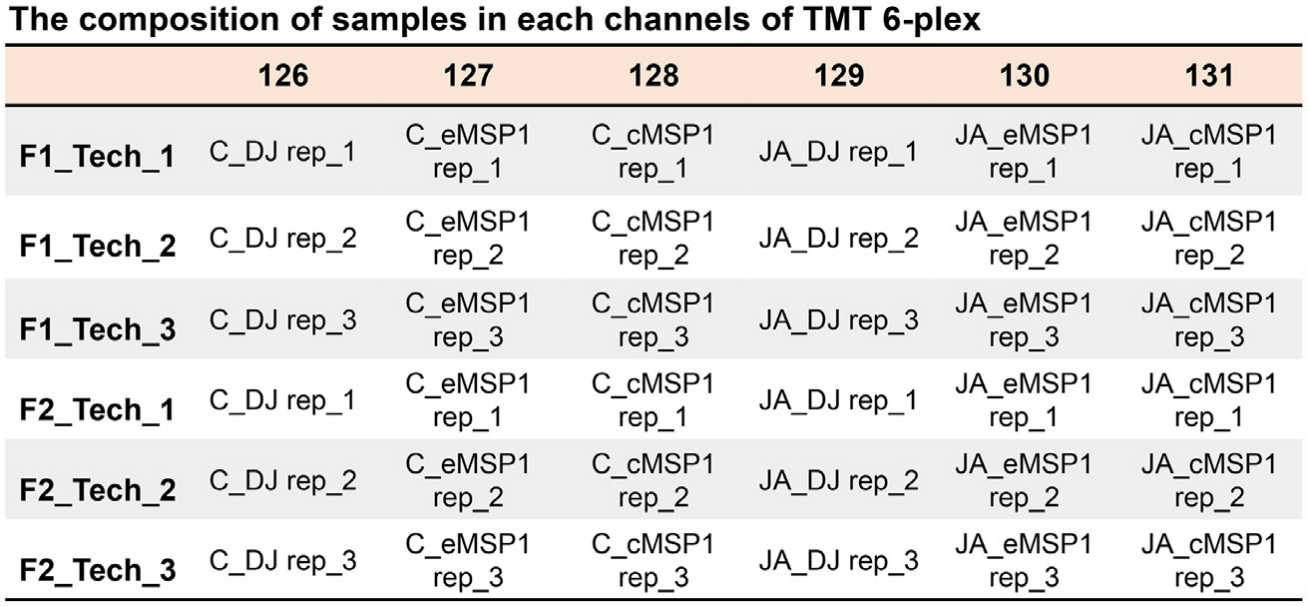
Table revealing the information of 6-plex TMT labeling using sample sets.

**Fig. 2:**
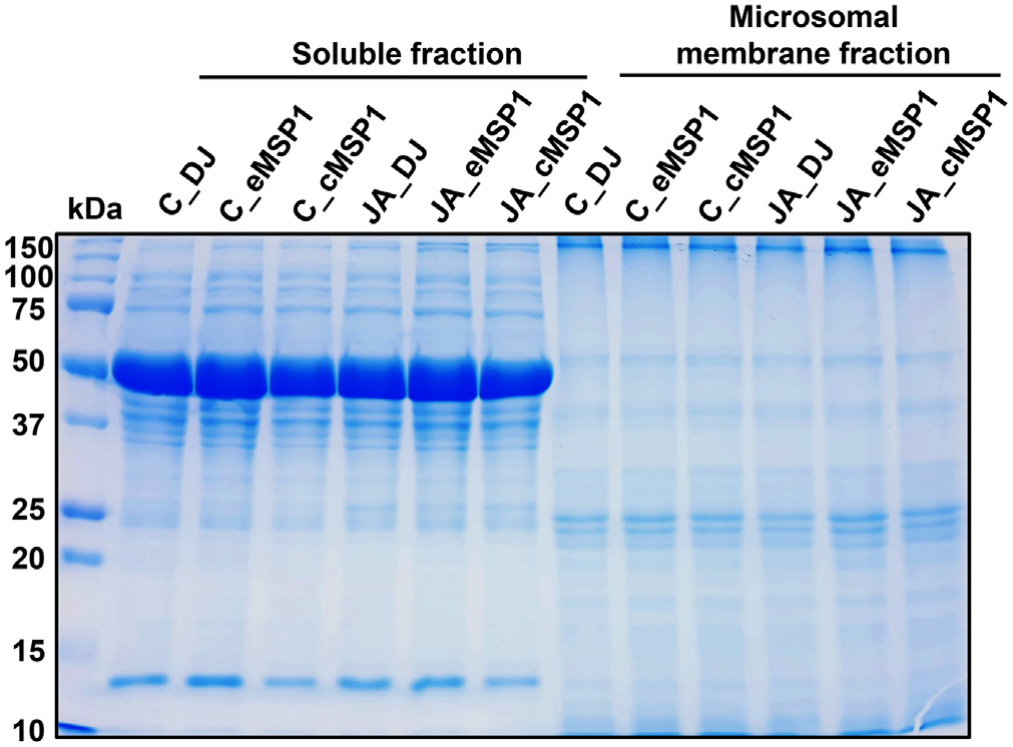
SDS-PAGE analysis of soluble and microsomal membrane proteins extracted by MME method.

**Fig. 3:**
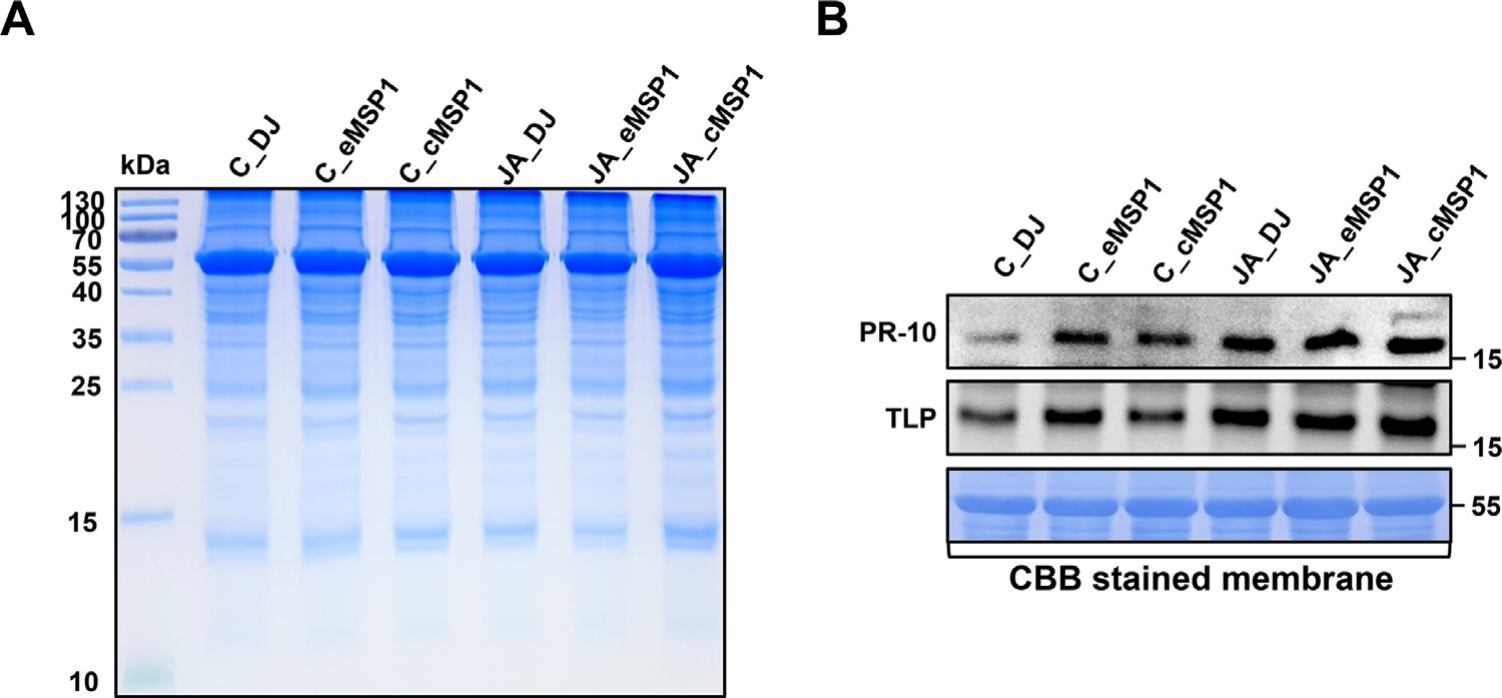
Western blot analysis of PTI signaling associated proteins, PR-10 and TLP, in DJ, eMSP1, and cMSP1 rice samples.

**Fig. 4:**
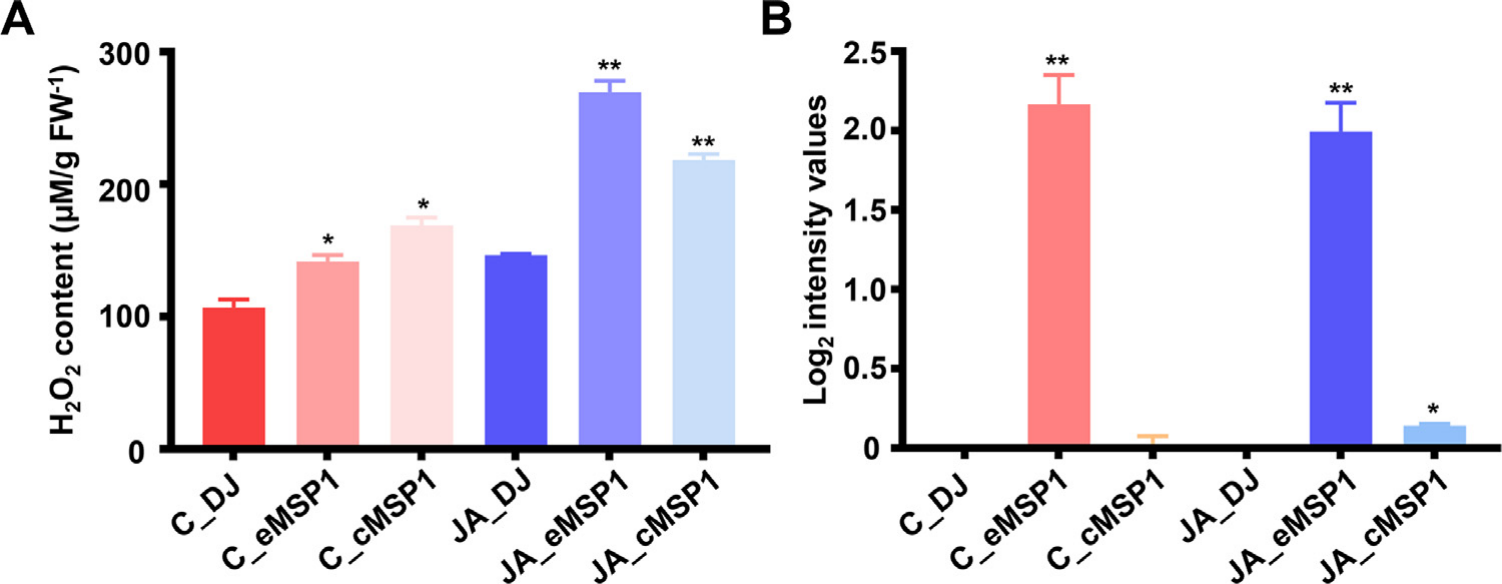
Biochemical assay for validation of physiological changes in response to expression of MSP1 in extracellular and cytoplasmic region by measuring (A) H_2_O_2_ contents. (B) Bar chart showing the accumulation profile of MSP1 protein particularly in C_eMSP1 and JA_eMSP1. (*) and (**) marks indicated significantly differences p-value < 0.05 and 0.001, respectively.

**Fig. 5:**
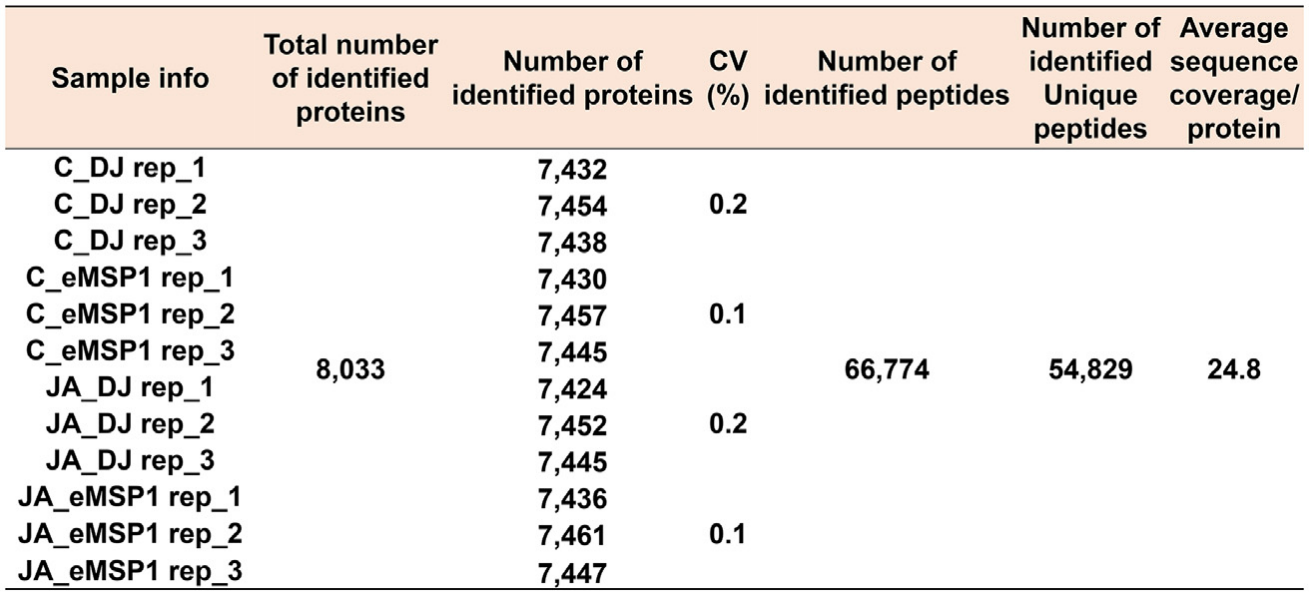
Overview of identified proteins in the three replicates of each sample set by TMT labeling and LC-MS/MS analysis.

**Fig. 6:**
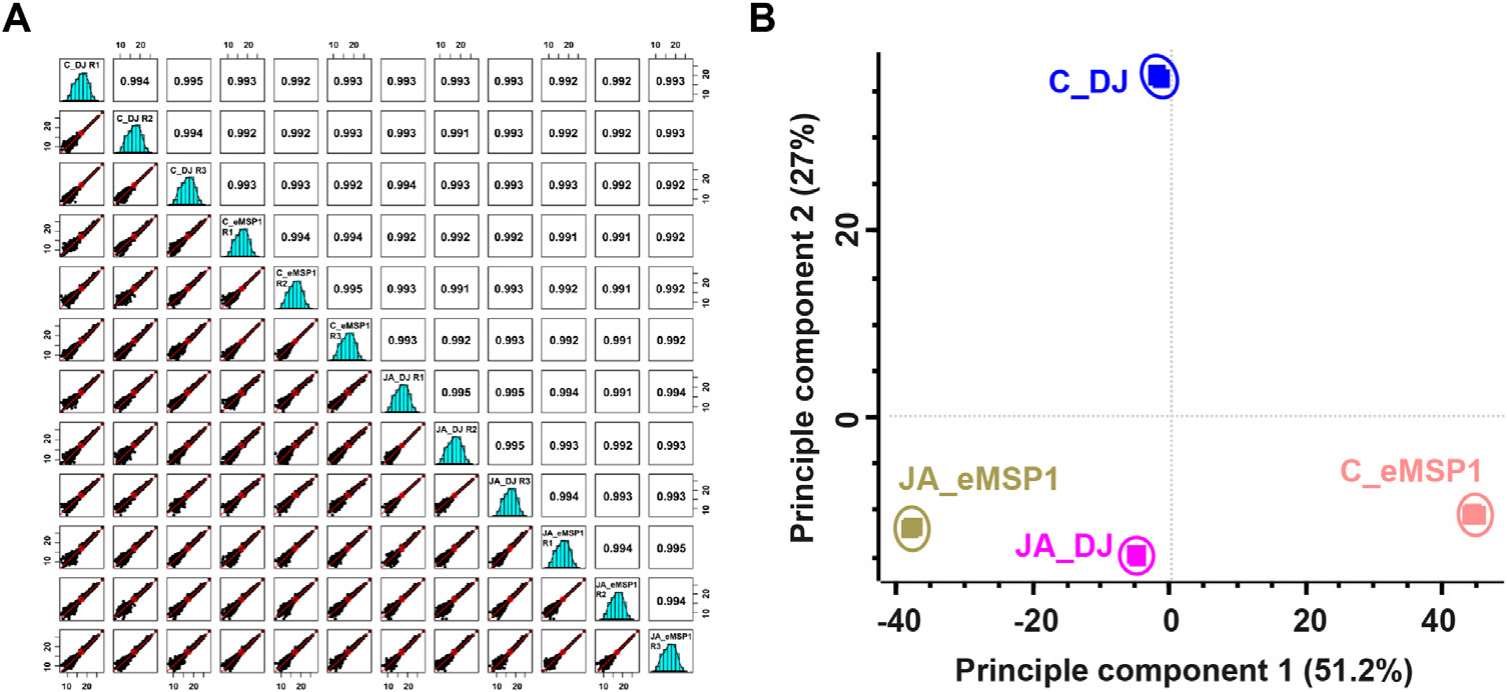
(A) Multi-scatter plot indicating the correlation among four samples. (B) Principal component analysis of 1,826 significant proteins identified by TMT labeling-based proteomic analysis.

The dataset reported here was obtained from the TMT-labeling based proteome analysis using DJ, eMSP1, and cMSP1 overexpressed rice cultivars with and without JA treatment (Fig. 1). Soluble and microsomal membrane proteins were isolated by a microsomal membrane enrichment (MME) method and the quality of the isolated proteins were first checked on SDS-PAGE (Fig. 2). In addition, Western blot analysis was carried out using pathogenesis related protein 10 (PR-10) and thaumatin like protein (TLP) antibodies for validation of samples (Fig. 3). Likewise, expression analysis of MSP1 confirmed expression of MSP1 transcript in eMSP1 and cMSP1 samples using following list of primers in Supplementary Table 1. Moreover, prior to the proteome analysis, the content of hydrogen peroxide (H_2_O_2_) was measured in each sample set to check for the MSP1 induced oxidative burst, a hallmark of PTI signaling (Fig. 4A). Further biochemical analysis showed degradation of MSP1 in cMSP1 sample in 4 weeks-old rice leaves, therefore, downstream analysis was performed without cMSP1 sample sets to reduce the complexity of proteome data (Fig. 4B). Isolated soluble and microsomal membrane proteins from DJ and eMSP1 samples were subjected to high-throughput proteome analysis using a TMT-based quantitative proteomics approach which led to the identification of a total of 8,033 proteins (Fig. 5). In addition, the application of internal reference scaling (IRS) normalization and sequential downstream statistical analysis (Benjamini–Hochberg FDR < 0.05 with a fold change of more than 1.25) revealed the identification of 1,826 significantly modulated proteins across the four sample sets (Supplementary Table 2). Multi-scatter plot and principal component analysis (PCA) were carried out using Perseus software to investigate the correlation and variation among different samples (Fig. 6). Furthermore, functional characterization of 1,826 significantly modulated proteins were performed using MapMan software (Supplementary Table 3 and 4).

## 3 Experimental Design, Materials and Methods

### 3.1 Plant Materials, RNA Isolation, and qRT-PCR Analysis

Seeds of wild-type (DJ) and MSP1 overexpression line (eMSP1) were sterilized with 0.05% spotak solution (Bayer Crop Science, Republic of Korea) and washed with distilled water five times. The sterilized seeds were allowed to germinate and transplanted to Yoshida solution [2] with and without supplementation of 250 μM JA. Total RNA was isolated from the leaves using TRIzol reagent (Invitrogen, Carlsbad, CA, U.S.A.) and cDNA was synthesized using QuantiNova Reverse Transcription Kit (Qiagen, Hilden, Germany). The qRT-PCR was carried out using Rotor-Gene Q real-time PCR cycler (Qiagen, Hilden, Germany) with QuantiNova SYBR Green Master Mix (Qiagen, Hilden, Germany) [3].

### 3.2 Extraction of Soluble and Microsomal Proteins using MME Method and In-Solution Trypsin Digestion

Extraction of soluble and microsomal membrane proteins from rice leaves was carried out using MME method as described before [4]. Briefly, 200 mg of fine ground leaf powder was homogenized with a high-density sucrose (HSD) buffer [37.5 mM HEPES, pH 8.0, 37.5% (w/w 1.215 M) sucrose, 7.5% (v/v) glycerol, 15 mM EDTA, 15 mM EGTA, 1 mM DTT, 100x Halt^TM^ protease inhibitor cocktail (Thermo Scientific, MA, USA)] and then clear supernatant was collected after centrifugation at 600 g for 3 min at 4 °C. Collected supernatant was diluted with ddH_2_O in 1:2 ratio of supernatant:ddH_2_O (v/v) and centrifuged at 21,000 g for 90 min at 4 °C. The supernatant so obtained after centrifugation was used as soluble proteins while the pellet contained microsomal proteins that were resuspended in wash buffer [20 mM HEPES, pH 8.0, 5 mM EDTA, 5 mM EGTA] and re-centrifuged at 21,000 g for 45 min at 4 °C. The soluble protein fraction was precipitated with 4 volumes of 12.5% TCA/acetone containing 0.07% (v/v) *β*-mercaptoethanol. Finally, precipitated microsomal and soluble proteins were washed with 80% acetone containing 0.07% (v/v) *β*-mercaptoethanol [1] and used for in-solution trypsin digestion by filter-aided sample preparation (FASP) method [5,6]. In brief, acetone precipitated proteins (300 μg) were dissolved in denaturation buffer [4% SDS and 100 mM DTT in 100 mM TEAB, pH 8.5], sonicated for 3 min, and incubated at 99 °C for 30 min. The denatured proteins were then diluted 10 times with urea buffer [8 M urea in 100 mM TEAB, pH 8.5] and loaded on a 30K spin filter (Merck Millipore, Darmstadt, Germany). SDS was subsequently removed by buffer exchange with urea buffer and cysteine alkylation was accomplished through the addition of alkylation buffer [50 mM iodoacetamide (IAA) in 100 mM TEAB, pH 8.5] [5,6]. After alkylation, trypsin solution dissolved in 5% ACN (enzyme-to-substrate ratio [w/w] of 1:100) was added and incubated at 37 °C for overnight. The concentration of digested peptides was measured using the Pierce Quantitative Fluorometric Peptide Assay kit (Thermo Scientific, MA, USA) following the manufacturer's instructions.

### 3.3 TMT-Labeling Based Proteomic Analysis with QExactive MS

TMT labeling of the digested peptides was carried out using TMT-6plex kit following manufacturer's protocol [1,5]. Briefly, each TMT reagent was re-suspended in 120 μL of anhydrous ACN and subsequently 25 μL of each TMT reagent was added in samples. The reaction was quenched by the addition of hydroxylamine to a final concentration as 0.3% (v/v) and the labeled samples were equally combined and desalted using the HLB OASIS cartridge. Desalted peptides were re-constituted in loading solution [15 mM Ammonium formate and 20% ACN] and fractionated into 12 fractions by basic pH reversed phase (BPRP) chromatography using in-house developed stage-tips, prepared with C18 Empore disk membrane (3M, Bracknell, UK) at the bottom and POROS 20 R2 reversed phase resin [6], [7].

The fractionated peptides were dissolved in solvent-A (2% ACN and 0.1% formic acid) and separated by reversed-phase chromatography using a UHPLC Dionex UltiMate^TM^ 3000 instrument (Thermo Fisher Scientific, MA, USA). The two-column system with a trap column column (Thermo Scientific, Acclaim PepMap 100 trap column, 100 μm×2 cm, nanoViper C18, 5 μm, 100 Å) and analytic column (Thermo Scientific, Acclaim PepMap 100 capillary column, 75 μm×15 cm, nanoViper C18, 3 μm, 100 Å) were carried out for separation of peptides. The peptides were separated with 150 min of nonlinear gradient from 2% to 35% solvent-B (100% ACN and 0.1% formic acid), 40 to 95% for 10 min, followed by 95% solvent-B for 5 min and 2% solvent-B for 15 min, respectively, as described before [1]. Electrospray ionization source was coupled with quadrupole-based mass spectrometer QExactive™ Orbitrap High-Resolution Mass Spectrometer (Thermo Fisher Scientific, MA, USA). Resulting peptides were electro-sprayed through a coated silica emitted tip (Scientific Instrument Service, NJ, Amwell Township, USA) at an ion spray voltage of 2,000 eV. The mass spectra were measured in a data-dependent mode for the 15 most abundance peaks (Top15 method). The precursor ions were acquired with a resolution of 70,000 at 200 m/z in a mass range of 350–1,800 m/z. The automatic gain control (AGC) target value was with 3×10^6^ and the isolation window for MS/MS was 1.2 m/z. Ion activation/dissociation with Higher Energy C-trap Dissociation (HCD) scans were acquired at a resolution of 35,000 and 32 normalized collision energy (NCE). The AGC target value for MS/MS was 2×10^5^. The maximum ion injection time for the survey scan and MS/MS scan was 30 ms and 120 ms, respectively. The proteomics data were deposited to the ProteomeXchange Consortium *via* the PRIDE partner repository with the dataset identifier PXD032689 [1,8].

### 3.4 Data Analysis and Functional Classification

The acquired raw mass spectrometry data were analyzed using MaxQuant software (ver. 1.6.17.0) integrated Andromeda search engine [9]. The obtained MS/MS spectra were cross-referenced against the Phytozome *Oryza sativa* database (MSU v6.0, 67,393 entries) using MaxQuant software with integrated Andromeda search engine. The TMT data was processed using default precursor mass tolerances as 20 ppm for the first search and 4.5 ppm for the main search with reporter mass tolerance set to minimum as 0.003 Da and the minimum 0.5 reporter precursor ion fraction (PIF), respectively. The TMT data was searched based on 0.5 Da of a product mass tolerance with a maximum of two missed tryptic digestions. Carbamidomethylation of cysteine residues was specified as fixed modification. Furthermore, acetylation of lysine residues and oxidation of methionine residues were selected as variable modifications. The false discovery rate (FDR), set at 1% for peptide identifications, was determined based on a reverse nonsense version of the original database. The normalization of reporter ion intensities was performed by IRS normalization method using R studio as described before [1,5]. Further downstream data processing and the statistical test were performed by the Perseus software (ver. 1.6.15.0) [10]. The missing value imputation of reporter ion intensities was carried out by calculation of normal distribution (width: 0.3, downshift: 1.8) using Perseus software. Multiple sample test analysis was performed to identify the statistically significant differences in the protein abundance (multiple ANOVA test controlled by a Benjamini–Hochberg FDR threshold of 0.05, ≥ 1.25-fold change). Significantly modulated proteins were functionally annotated using MapMan software.

## Ethics Statements

No animal experiments were performed in this study.

## CRediT authorship contribution statement

**Cheol Woo Min:** Conceptualization, Methodology, Software, Writing – original draft, Writing – review & editing. **Jeong Woo Jang:** Writing – original draft, Methodology. **Gi Hyun Lee:** Methodology. **Ravi Gupta:** Methodology, Supervision, Writing – review & editing. **Sun Tae Kim:** Supervision, Funding acquisition, Writing – review & editing.

## Declaration of Competing Interest

The authors declare that they have no known competing financial interests or personal relationships that could have appeared to influence the work reported in this paper.

## Data Availability

TMT-based quantitative proteome data of MSP1 overexpressed rice (Original data) (DIB).
